# Optimal Strategy for Competence Differentiation in Bacteria

**DOI:** 10.1371/journal.pgen.1001108

**Published:** 2010-09-09

**Authors:** C. Scott Wylie, Aaron D. Trout, David A. Kessler, Herbert Levine

**Affiliations:** 1 Center For Theoretical Biological Physics, University of California San Diego, La Jolla, California, United States of America; 2 Department of Mathematics, Chatham University, Pittsburgh, Pennsylvania, United States of America; 3 Department of Physics, Bar-Ilan University, Ramat-Gan, Israel; University of Toronto, Canada

## Abstract

A phylogenetically diverse subset of bacterial species are naturally competent for transformation by DNA. Transformation entails recombination of genes between different lineages, representing a form of bacterial sex that increases standing genetic variation. We first assess whether homologous recombination by transformation is favored by evolution. Using stochastic population genetic computer simulations in which beneficial and deleterious mutations occur at many loci throughout the whole genome, we find that transformation can increase both the rate of adaptive evolution and the equilibrium level of fitness. Secondly, motivated by experimental observations of *Bacillus subtilis*, we assume that competence additionally entails a weak persister phenotype, i.e., the rates of birth and death are reduced for these cells. Consequently, persisters evolve more slowly than non-persisters. We show via simulation that strains which stochastically switch into and out of the competent phenotype are evolutionarily favored over strains that express only a single phenotype. Our model's simplicity enables us to derive and numerically solve a system of finite-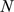 deterministic equations that describe the evolutionary dynamics. The observed tradeoff between the benefit of recombination and the cost of persistence may explain the previously mysterious observation that only a fractional subpopulation of *B. subtilis* cells express competence. More generally, this work demonstrates that population genetic forces can give rise to phenotypic diversity even in an unchanging and homogeneous environment.

## Author Summary

In certain environmental conditions, populations of the bacterium *Bacillus subtilis* split into two physiologically distinct phenotypes. While some cells continue to grow and divide, a minority become “competent” for transformation by extracellular DNA. This differentiation process is driven not by genetic differences among cells, but rather by noisy molecular fluctuations. Although the differentiation process is thought to confer an evolutionary advantage, the basis of this advantage has remained elusive until now. We developed computer simulations of the joint dynamics of cell replication, cell death, mutation, and the quasi-sexual transfer of genes through the extracellular DNA pool. We find that bacterial sex via DNA transformation is indirectly favored by evolutionary forces. However, the indirect benefits of sex are counterbalanced by a reduced replication rate. We find that these opposing forces present an evolutionary dilemma best solved when the population splits into the two experimentally observed phenotypes. These results present a mechanism that selects for phenotypic diversity, even in an unchanging and homogeneous environment.

## Introduction

Bacteria mainly reproduce asexually, which has strong implications for the degree and patterns of intraspecific genetic diversity. However, three quasi-sexual mechanisms operate to combine genetic information between different lineages: conjugation, transduction, and transformation. Among these, transformation is unique in that the genes responsible for it are natively present on the chromosome, suggesting that it is favored by natural selection. Cells capable of this act are said to be competent for genetic transformation, or “competent” for short. In this article, we consider only natural competence, as opposed to that induced artificially in the laboratory by electroporation, etc. For a review of competence in bacteria, see [1] and references therein.

The source of extracellular DNA during transformation in wild populations is not entirely clear. Detritus from cell lysis probably contributes to this pool, although active secretion from intact cells is also a possibility [2]. Perhaps more importantly, extracellular DNA can originate from the same or from different species. However, sequence similarity between the host chromosome and the incoming fragment increases the probability of integration [1]. This suggests that homologous gene recombination (HGR) of DNA from conspecifics occurs more often than horizontal transfer of novel genes between species. Although interspecific transfer is known to play an important role in microbial evolution [3], here we focus exclusively on *homologous* recombination (HGR).

Besides transformation of DNA, a secondary property of competence observed in *Bacillus subtilis* is reduced rates of metabolic activity [4] and cell division [5], [6]. The increased time between cell divisions may be necessary to perform the chromosomal manipulations required for HGR without causing DNA damage [5]. Furthermore, perhaps *because* of reduced metabolic rates, competent *B. subtilis* cells also die more slowly when exposed to antibiotics, as compared to non-competent cells [5]–[7]. Reduced birth and death are the hallmark of the “persistence” phenotype [8], [9]. In *E. coli*, persisters are known to stochastically switch back and forth from the usual growth (i.e. “vegetative”) state [10]. Following recent work by Johnsen et al. [6], we describe competence in terms of both recombination and persistence.

With few exceptions, competence is regulated in naturally transformable species [11]. Here, we focus on the most thoroughly studied example, *B. subtilis*, in which competence is considered a stress response [12]. Although competence in this species is just one aspect of a more complicated survival strategy, notably including sporulation, here we focus exclusively on competence. Under normal laboratory conditions (e.g. growth in LB broth), expression of competence genes or their associated phenotype cannot be detected. However, certain “competence media” [13] induce noisy activation by the regulatory circuit and a *differentiation* process in which merely 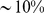 of cells express competence while the remaining 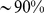 continue vegetative growth or perhaps sporulate [11]. There are no known conditions that induce all cells to simultaneously become competent in *B. subtilis*. Furthermore, recent single cell experiments dramatically show that this 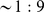 ratio is a *dynamic equilibrium*: over timescales 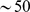 hours, a single cell lineage may enter, exit, and then re-enter competence [14]–[16]. As those authors note, the statistics of competence initiation are consistent with a simple memory-less model of phenotypic switching. The observed phenotypic differentiation originates not from genetic differences, but rather from noisy fluctuations in the key transcription factor 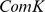 [14]–[18].

Here, we ask “why” only a fraction of *B. subtilis* cells become competent, i.e. why the population exhibits phenotypic diversity. Previous studies [10], [19]–[21] interpret phenotypic diversity as “bet hedging” against an uncertain, fluctuating environment. By contrast, in this article we demonstrate that phenotypic diversity for the competence phenotype can result from natural selection even in an unchanging, homogeneous environment. The fact that competence is intimately related to the ability to create genetic changes suggests that the road to understanding this mysterious phenotypic diversity goes through population genetics/evolutionary dynamics.

To this end, we developed evolutionary computer simulations that include both aspects of competence: HGR and persistence. Our *in silico* populations consist of an approximately constant number of vegetative and competent cells. HGR is not assigned an *a priori* advantage, but it turns out to be evolutionarily favored for indirect reasons related to the evolution of sex and recombination. These findings can be readily understood in relation to previous studies of HGR [22]–[25] (see Discussion for elaboration). Similarly to HGR, persistence is not assigned an *a priori* fitness effect and is, to a first approximation, evolutionarily neutral in our simulations. However, a closer analysis reveals that persistence incurs an indirect cost during adaptive evolution. We conclude that populations face a tradeoff when “deciding” what fraction of cells express competence (HGR is “good,” but persistence is “bad”). An alternative interpretation of this decision is that (lineages of) cells must decide how to allocate their time spent between the competent and vegetative phenotypes. During competence, novel recombinant genotypes are created by HGR, but these recombinants maximize their evolutionary success when they are later expressed in rapidly growing vegetative cells. This tradeoff could plausibly explain the phenomenon of heterogeneous competence expression in *B. subtilis*.

## Methods

### Genome Model

**Figure 1:**
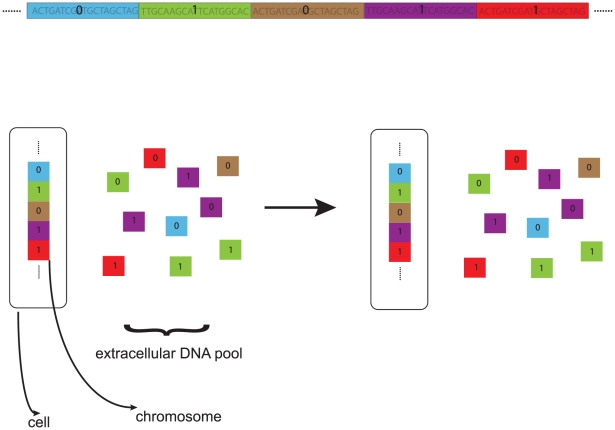
Genome and recombination model. **Each genome contains 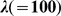 loci, each of which represents 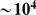 nucleotides and contributes independently to fitness.** We assume that two families of alleles are segregating at each locus: more fit (labeled by “one”) and less fit (labeled by “zero”). Upon recombination (HGR), the acceptor allele is replaced by a homologous (same color) donor allele drawn randomly from the extracellular DNA pool. The allele frequencies in the extracellular pool are assumed to be identical to those among the population of living cells. Thus, HGR is not *directly* favored. The genetic change is non-reciprocal: the acceptor allele is presumed degraded and not placed in the extracellular pool. For simplicity, we do not represent loci that enable HGR. In figure S1 and text S1, we briefly consider an extracellular pool loaded with excess deleterious mutations.

We model the bacterial chromosome as 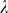 loci 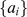, each of which has either a more fit (one) or less fit (zero) allele (figure 1). For simplicity, we do not represent the genes responsible for competence, nor do we allow mutations to change a cell's competence properties.

A cell's intrinsic birth rate (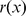) simply equals the fraction of “ones” in the genome
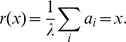
(1)For our continuous time dynamics (see below), the additive structure of equation 1 corresponds to independent contributions across loci. If discrete (e.g. Wright-Fisher) dynamics were used instead, then a multiplicative function would correspond to independent loci. Synergistic or antagonistic epistasis can easily be included by choosing a different functional form of 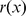. The carrying capacity 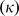 represents limited nutrients and/or space, which decreases the actual birth rate (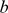) from the intrinsic value (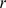):
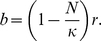
(2)


### Simulation Dynamics

In continuous time (overlapping generations), one of the following simulation steps is *stochastically* chosen according to the well known “Gillespie algorithm” [26]:

1. Phenotypic transition, with rates 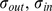 per cell.
2. Replication, with rate per cell equal to 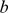 (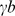) for vegetative (competent) cells.
3. Mutation: Upon replication, each “one” undergoes a deleterious mutation (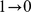) with probability 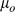, and each “zero” undergoes a beneficial mutation (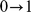) with probability 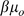.
4. Death: A vegetative (competent) cell is annihilated with per cell rate 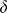 (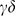).
5. Recombination: A competent cell undergoes a transformation-like event with rate 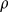 per competent cell (figure 1).

C++ code for our simulations is available upon request.

### Recombination Model

Homologous recombination by transformation (HGR) occurs with rate 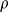 between a living cell (the acceptor) and a pool of extracellular DNA (the donor) derived from recently lysed conspecific cells. The allele at (exactly) one randomly chosen locus in the acceptor is replaced by a homologous allele chosen randomly from the extracellular pool. Since, for simplicity, we do not explicitly represent the genes that enable recombination, these genes obviously cannot be transferred by an HGR event in our model. This implies that a recombining cell cannot transform itself into a non-recombining cell in our model. Further, for simplicity, the allele frequencies in the extracellular pool are assumed to be identical to those in the population of living cells. This assumption may not be true for real populations because cells carrying deleterious/lethal mutations could lyse more often than fit cells (see figure S1 and text S1). Unlike other models [6], [23], we do not explicitly consider how 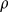 depends on population density (cells per volume). Rather, we assume that density is constant regardless of the census size, and lump density effects within the parameter 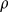 (see parameter estimation below).

### Velocity Measurements

For “shuffled” and clonal initial conditions, populations were founded by cells all having the same number of “ones” (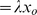) in their genomes. The initial number of cells was chosen as 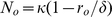 so that 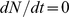, i.e. so that the population as a whole was neither growing nor shrinking. For “shuffled” initial conditions, the 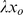 “ones” in each genome were independently assigned random positions in each founding cell, thus maximizing the genetic diversity consistent with fixed initial fitness. By contrast, each cell in the clonal initialization scheme had exactly the same genotype. The populations were then allowed to evolve up a significant portion of the fitness landscape (40 beneficial mutations for the data presented) so as to minimize the influence of initial conditions. We then calculated the average velocity during the interval in which the mean number of “ones” increased from 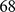 to 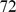. These velocities were averaged over either 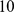 or 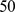 replicates (see figure captions).

For runs with “natural” initial conditions, the procedure was slightly more complicated. First, populations were “burned in” to an equilibrium configuration (see below) while recombining with rate (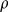). Next, 70 of the 100 loci were chosen randomly. Each cell then had its allele “flipped” at each of these 70 loci. This had the effect of reducing the number of “ones” in each genome by an amount ≲70, while maintaining the level of genetic diversity obtained during a long period in equilibrium. This is equivalent to a sudden change in the fitness function (so that many loci which were beneficial (deleterious) under the old fitness function are deleterious (beneficial) under the new fitness function) that would result from a sudden environmental change. After this complicated initialization step, 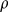 was unchanged from its value during the “burn in” phase, and the procedure was identical to that for the clonal and shuffled cases outlined above. One of 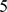 “burn in” populations was randomly chosen for the initialization procedure for each of the 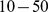 replicate velocity measurements.

### Equilibrium Populations

Populations were founded with a clone having a perfect genome (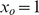) and a given recombination rate 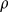. The initial number of cells was 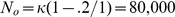. The simulation dynamics then proceeded for 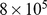 time units, which, for 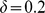, and 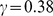 corresponds to approximately 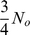 “generations,” i.e. 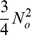 birth events. Since 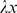 had clearly reached equilibrium (figure 3,left) and 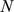 generations is known to be a very long population genetic timescale, we have reasonable confidence that these populations reached an equilibrium level of genetic diversity. The equilibrium fraction of “ones” in figure 3,right was determined by averaging over the final 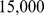 generations and 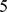 replicate trials.

### Competitions

We performed competitions both when the resident was adapting up the fitness peak and also when it had reached mutation/selection/drift equilibrium. In the adapting case, we allowed 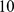 replicate populations to adapt from 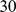 to 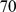 “ones,” starting from a single clone, then saved the population. For each competition, one of the 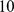 saved populations was randomly chosen, from which 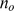 (between 1 and 100) cells were selected at random and changed to the invader type (either purely competent or stochastically switching). Thus, the new invaders occur in randomly sampled genetic backgrounds and compete in populations that contain a semi-natural level of diversity. The procedure for the equilibrium case was similar, but only 5 initial populations were saved, after first evolving for ≥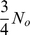 generations, as described above. Each data point in figure 5 was derived from at least 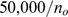 competitions. 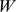 was computed by multiplying the slope of the least-squares linear fit by the average number of cells (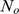) present when the invader was introduced.

### Parameter Estimation

Most of our simulations shared core set of parameters, which we now discuss and relate to experiment. The switching and growth parameters are based on data from [14], [15]. These authors observed a median cell division time of 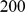 minutes, excluding competence events. In our constant population size scenario, this quantity must equal the death rate, so 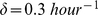. The mean duration of competence events was 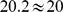 hours, i.e. 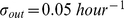. Based on the supplementary movies from [14], upon escape from competence, a cell fragments into 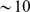 vegetative cells. Our model treats this sudden burst of offspring from a competent cell as continuous growth. Thus, 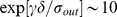, which implies that 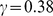. We used 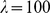 loci which each represent recombining segments of 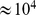 base pairs [1]. This implies a genome length of 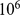 base pairs, which can be compared to 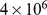 and 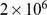 for *B. Subtilis* and *S. pneumoniae*, respectively.

We also note that competence initiation was observed during 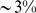 of vegetative cell division events [15], [16]. This implies that 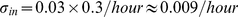. If the equilibrium fraction (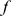) of competent cells depended only on the switching rates (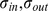), and not on selection for or against cells expressing competence, then 
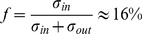
, in fair agreement with experimental observations. This is consistent with our quasi-neutral treatment of competence expressing cells.

Our simulations used idiosyncratic time units (TU) that can easily be related to hours. In all simulations, we used 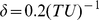, which can be equated to 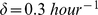, yielding the conversion factor 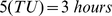. Multiplying those parameters values from simulations which have dimensions 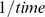 (see figure captions) by 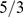 reproduces the experimental estimates above.

The deleterious mutation rates for *B. Subtilis* and *S. pneumoniae* are likely roughly similar to that of *E. coli*, which has been experimentally estimated as 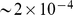 [27] per genome, which is 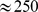 times smaller than our value of 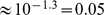. However, our value could plausibly correspond to a mutator strain (see, e.g. [28]) and thus is not entirely unrealistic. Experimental estimates of the beneficial mutation rate range widely from 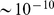 [29] to 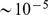 [30] in *E. coli*, depending on the environmental conditions and which mutations, exactly, are being measured. Thus, 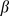, which equals the ratio of beneficial to deleterious mutation rates, experimentally lies in the range 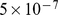 to 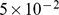. Our value of 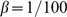 is thus on the high side, but not unreasonable. See figures S4,S5,S10 for some results using 100-fold lower mutation rates.

Our reason for using such large mutation rates is that we are interested in the regime in which many beneficial mutations are spreading simultaneously, which occurs when 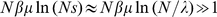 [31]. This is a plausible biological scenario because real microbial population sizes can easily exceed 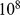. However, this is prohibitively large for simulations like ours which are fully stochastic and must potentially track any of 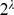 possible genomic variants. Thus, our only recourse is to make 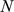 as large as computationally feasible, 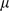 as small as possible, and 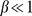, while keeping 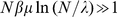. We believe that our choices of population size, 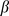, and 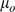 are reasonable for these purposes, although they are certainly not equivalent to actual biological parameters. Our assertion that they capture the qualitative behavior of the real, experimental parameter set is bolstered by solutions to the finite-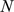 deterministic model (see figure S9), which can handle arbitrary parameters values. Furthermore, in figures S4, S5, S10 we explore some consequences of using biologically realistic, 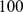 fold lower mutation rates in simulations.

To the best of our knowledge, the recombination rate relevant to our model has not been directly measured. However, a related estimate was recently obtained for *B. subtilis* by fitting a model of bacterial growth and transformation to the experimentally observed kinetics of transformation for a particular antibiotic resistance marker [6] with partial homology to the acceptor strand. In order to quantitatively relate our recombination parameter 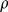 with theirs (call it 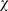), it is necessary to discuss three important differences between the two models. First, those authors modeled transformation with second order kinetics, such that the total rate of recombination in the population equals 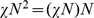. By contrast, our description uses first order kinetics, such that the overall rate of recombination equals 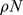. This suggests an identification of 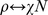, where 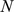 is the population size in their experiments. However, a second difference between our description and theirs is that they only observe transformants at a particular antibiotic resistance locus, whereas our model refers to each of the 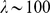 loci throughout the genome. Thus, using their values of 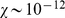 and 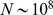, we obtain 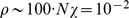. In relation to the total mutation rate per genome (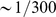 per cell division [32]), the results from [6] imply 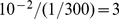 recombination events per mutation event. This value roughly agrees with estimates derived from multi-locus sequence typing (MLST) analyses of several bacterial species including *S. pneumoniae* (e.g. [33], [34]. MLST estimates are obtained by comparing the number of *fixed substitutions* among populations that likely arose via mutation to those that likely arose via recombination. Since these two kinds of genetic changes probably achieve fixation with different probabilities, the MLST approach does not directly reflect our parameter 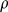. Nevertheless, those studies were designed to minimize this effect by examining only housekeeping genes, and their estimates serve as a valuable experimental reference point. In any case, both the MLST approach and that adopted by [6] likely underestimate the true recombination rate because they cannot detect “null” exchanges among members of the same local population, in which the donor and acceptor alleles are identical. Thus, the experimental value of our parameter 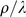 is probably somewhat larger than 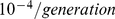. Given our values for the remaining set of parameters, in order for phenotypic switching to be optimal (or, for that matter, for HGR to have a significant effect) during adaptation requires 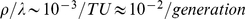. This certainly stretches experimental bounds but is not entirely implausible. However, according to figure 3, HGR can confer increased equilibrium fitness even for 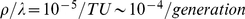.

## Results

We constructed fully stochastic, multi-locus computer simulations of finite populations in which deleterious and beneficial mutations of small effect (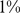) occur throughout the entire genome. Birth and death occur independently, and total population size (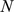) is controlled logistically by a carrying capacity 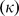. Each locus represents 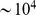 nucleotides, which is the approximate length of (single stranded) DNA fragments incorporated by *B. subtilis* and *S. pneumoniae* during a natural transformation event [1]. Recombination (HGR) events correspond to the replacement of a single locus in a living cell by a homologous allele drawn from an extracellular DNA pool (figure 1). At each locus, the distribution of alleles in the extracellular pool is exactly the same as in living cells. Thus, there is no *direct* advantage to HGR. The effects of random genetic drift and linkage between many loci are naturally included in our model.

We assume that each locus independently contributes toward organismal fitness, i.e. that there is no epistasis between loci. We approximate the fitness contribution of each locus as a binary variable: if it fully contributes, then it is assigned a “one,” otherwise it is assigned a “zero.” Neither zeros nor ones correspond to unique nucleotide sequences, but rather to entire families of 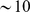 kilobase sequences that represent more fit (one) or less fit (zero) alleles. 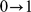 changes at a locus represent mutations that either unconditionally improve an organism's adaptation to its environment or else compensate for a deleterious mutation somewhere within that same locus. Likewise, 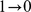 mutations, which occur much more frequently than do 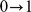, represent deleterious mutations. Organismal fitness can be defined in several ways. Regardless of the specific definition, fitness is clearly an increasing function of the fraction (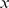) of “ones” in the genome and, for brevity, we will often simply refer to 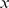 as fitness.

The reader is strongly encouraged to read Methods for important elaborations on the quantities and processes presented in this section. We begin by presenting the effects of HGR in populations consisting entirely of competent cells. We next build on these results and describe the interaction between HGR and persistence when cells stochastically switch between the competent and vegetative phenotypes.

### HGR Increases the Speed of Adaptation

Organisms are usually well adapted to their environment, i.e. their genomes reside near a local fitness peak in sequence space. However, if the environment suddenly changes, then the fitness peak can shift beneath the population, effectively displacing it to a slope in the fitness landscape. In this case, the population will likely evolve up this slope, toward a new locally optimal sequence. We first demonstrate that HGR can increase the speed of evolution up a smooth fitness peak, i.e. the speed of positive selection. For simplicity, we consider neither subsequent environmental changes nor the possibility that the population crosses a valley and jumps to a separate fitness peak [35].

**Figure 2:**
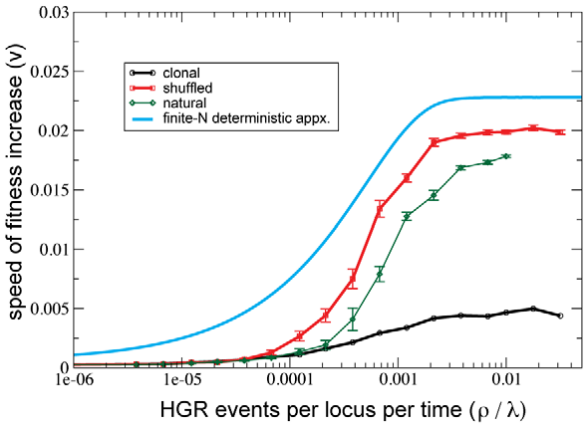
HGR accelerates adaptive evolution (i.e. positive selection) on a smooth fitness landscape. The initial level of genetic diversity is varied from high (“shuffled”) to medium (“natural”) to low (“clonal”). Shuffled populations initially contained sequences obtained by randomly placing 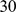 “ones,” whereas clonal populations initially contained one unique sequence. “Natural” initialization represents an abrupt displacement from mutation/selection/drift balance at the fitness peak. 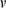 was measured during the interval in which the mean number of “ones” passed between 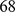 and 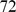. Averages were taken over 10 replicates. Error bars denote one standard error. Parameters are 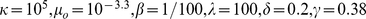.

Previous articles based on similar models [22], [23], [36], [37] have demonstrated that HGR increases the speed of adaptation up a smooth fitness peak. Figure 2 confirms and elaborates upon these findings in the context of our current model and parameter values. We see that the speed of adaptive evolution (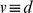
“*ones*”/*dt*) increases with the rate of HGR (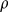) and eventually reaches an asymptotic value when there are 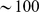 HGR events per mutation event (for these parameter values). The essential conclusion that HGR increases the speed of adaptation was also made experimentally in the case of transformation in *Helicobacter pylori* [38] and the distinct but conceptually similar mechanism of conjugation in *Escherichia coli* [39]. However, two other studies [40], [41] do not support this conclusion. See [23] for further discussion and interpretation of these experiments.

The influence of HGR depends crucially on the population's level of genetic diversity. To explore how initial conditions impact this effect, we initialized our simulations with varying levels of diversity (figure 2). When populations were founded by a single clone, 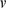 was relatively small. On the opposite extreme, we also founded populations with many randomly generated sequences compatible with the initial fitness level, which resulted in a much larger 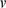. As a third, perhaps more realistic alternative, we initialized simulations by suddenly displacing a population in mutation/selection/drift equilibrium from the fitness peak (see Methods). These “natural” initial conditions resulted in an intermediate level of diversity and a correspondingly intermediate rate of adaptation (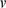). This “natural” level of diversity depends in an unknown and complicated way on the population size 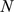, the mutation rate, and the fitness effect of mutations. The observed influence of initial conditions on the speed of adaptation is likely due to a long transient period during which neutral genetic diversity accumulates. Although computationally inconvenient, this transient is likely important in laboratory experiments and natural populations whose size fluctuates.

In figures S2, S3, S4 we explore how the aforementioned benefit of HGR depends on various parameters including population size, strength of mutations, and mutation rate. Generally, we find that HGR accelerates adaptation over a broad range of parameters. However, the magnitude of the advantage is reduced in smaller populations with low mutation rates. Conceptually, this is because these conditions decrease the genetic diversity upon which HGR acts. In fact, we see that under those conditions, increasing the level of HGR to extremely high levels can begin to *decrease* the rate of adaptation. The likely explanation for this effect is related to the non-reciprocal nature of bacterial transformation– a unique beneficial mutation can be lost by an HGR event at that locus (figures S2, S3, S4).

### HGR Can Increase Equilibrium Fitness

**Figure 3:**
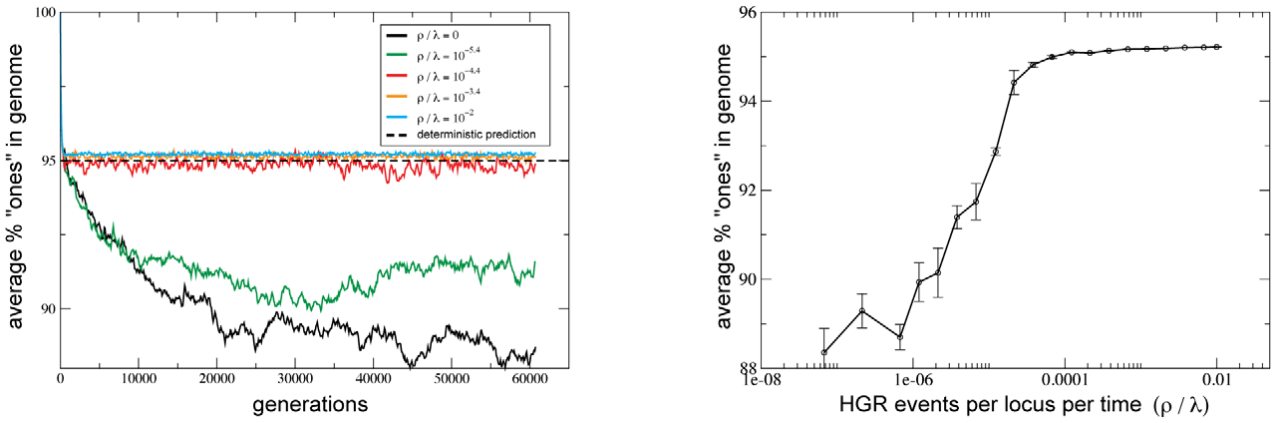
HGR increases equilibrium fitness. Recurrent deleterious mutations are predicted to decrease the number of ones by 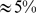 in infinitely large populations. In these finite populations we see an additional cost of 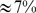 when HGR is absent. HGR restores the average fraction of “ones” to the deterministic (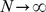) prediction (see main text). Averages were taken over the final 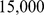 generations and 5 replicates. Error bars denote one standard error. Parameters are the same as in figure 2. See figure S5 for behavior with lower mutation rates.

Besides the rate of adaptation 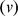, evolutionary success can also be measured by the fitness level achieved in mutation/selection/drift balance [42]. In order to explore this effect we founded populations with a perfectly adapted clone, which was then evolved for 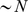 generations (figure 3). The average fitness declined initially due to the overwhelming number of deleterious mutations, reflecting the mutational load, the “fixed drift load” [43], [44], and a limited form of “Muller's ratchet” [45]. As deleterious mutations accumulated, more beneficial mutations became available, eventually leading to an equilibrium fitness level. This level increases monotonically with 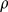, eventually reaching an asymptotic value when there are 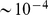 HGR events per locus per time, corresponding to 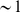 HGR event per mutation event. Thus, the rate of HGR needed to maximize equilibrium fitness is 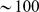 times less than that required to maximize the rate of adaptive evolution (for these parameter values).

Our measured equilibrium fitness should be compared to the standard deterministic (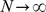) calculation [46] which neglects beneficial mutations. That classical method predicts that the number of “zeros” carried by a genome follows a Poisson distribution with mean 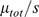, where 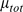 is the mutation rate per genome and 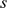 is the selection coefficient of (deleterious) mutations. For the parameters in figure 3 (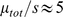), this formula implies that 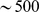 cells will be perfectly fit and that the mean fitness is 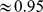. In stark contrast, figure 3 shows that, if HGR is not strong enough, stochastic fluctuations and finite population size reduce equilibrium fitness below 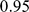 by an additional 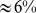. For higher levels of HGR, populations achieve, but do not substantially exceed, the deterministic prediction (the tiny excess beyond 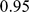 seen for large 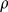 is likely due to the presence of back mutations in our model). These findings agree with and extend previous theoretical work [24], [25], [47] which showed that, when epistasis is absent, as it is in our model, recombination cannot increase equilibrium mean fitness *beyond the deterministic prediction*. However, figure 3 clearly shows that HGR can increase equilibrium fitness *beyond that which is actually achievable* in finite, noisy populations. This finding is broadly and conceptually important because it shows that epistasis is not necessary for HGR to confer an equilibrium advantage to moderately sized (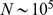 for our parameters) populations, in agreement with recent theoretical studies oriented toward eukaryotes [48]–[50].

Importantly, the benefits of HGR on equilibrium fitness are parameter dependent. For example, when we lowered the mutation rate by a factor of 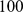 and kept 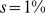, mean fitness in moderate size populations was independent of HGR and well described by the classical theory (see figure S5). We expect that the equilibrium benefits of HGR will be greatest in small populations with relatively high mutation rates and weak selection coefficients (so that 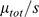 is large). Quantifying these dependencies remains a challenge for future work.

### Model of Persistence

As mentioned in the Introduction, competence is characterized by two distinct properties: recombination and persistence. Having presented our preliminary results showing the effects of HGR, we now turn our attention to persistence. We assume that when a cell switches to the persister phenotype, the reduction in birth rate is accompanied by a proportional reduction in death rate. In terms of our parameters, competent cells have

1. a non-zero rate of recombination (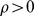)
2. replication rates reduced by a factor 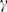 compared to vegetative cells 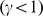

3. death rates also reduced by the factor 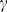 compared to vegetative cells 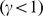

The second and third properties taken together comprise persistence. Traditional population genetics models, such Wright-Fisher sampling [51] or Moran's process [52], require that all individuals have the same death rate and thus these models cannot easily model persistence. This is why we resorted to a less traditional logistic model in which birth and death are decoupled (see Methods).

Ignoring recombination and mutation for the moment, under growth conditions in which the total number of cells is increasing (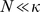), the frequency of persister cells will decline exponentially with rate proportional to 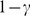. By contrast, when the total number of cells is decreasing under severe stress conditions (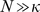), the frequency of persister cells will rise exponentially. In conditions intermediate between boom and bust, when the population size is stable, the frequency of persisters will remain approximately constant as long as their birth and death rates are each reduced *by the same factor* 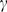. These dynamics can be thought of as a form of “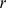 vs. 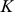 selection” [53], [54]. For a comprehensive treatment of these dynamics, see [55]–[57]. See Discussion for a comparison between ours and previous models of persistence.

Before proceeding, it is important to point out some consequences of this persistence model. As mentioned above, our persisters are competitively neutral compared to vegetative cells (though see [56] for a small stochastic correction). However, the situation becomes more subtle when spontaneous mutations are considered. Since most mutations occur during DNA synthesis and cell replication, the mutation rate of persisters is reduced by a factor 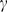. Additionally, beneficial (deleterious) mutations will expand (decline) more rapidly by a factor 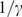 when expressed in vegetative cells. In other words, selection operates more quickly on vegetative cells than competent cells because selection is ultimately a consequence of birth and death events, both of which occur 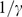 times more frequently among vegetative cells. Indeed, birth, mutation, and death, and therefore *the entire asexual dynamics*, *are all reduced by the common factor* 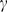, effectively slowing down time for persisters. These effects follow from persister cells' increased generation time, and they combine to impose an indirect cost to the persister phenotype *during periods of adaptive evolution*.

### Phenotypic Switching between Competent and Vegetative Phenotypes Can Optimize the Speed of Adaptive Evolution

**Figure 4:**
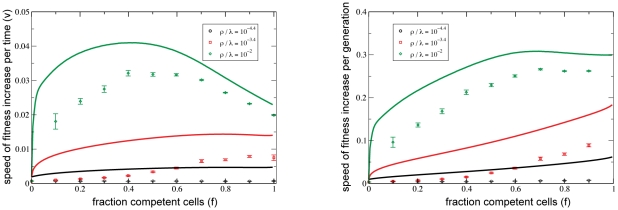
Optimal mixed strategy for competence. If HGR is strong enough, the rate of adaptation (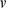) is maximized when only a finite fraction (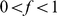) of cells express competence at any moment (left). Competent populations are favored because they perform HGR, but disfavored because of their longer generation time. This latter effect is normalized out by considering the speed of evolution *per generation*(right). The tradeoff then disappears and 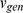 is maximized by a purely competent population. However, 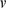 (and not 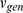), is the evolutionarily relevant measure. Phenotypic diversity is maintained via the switching rates 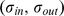: The fraction of cells expressing competence at any moment was adjusted by varying the rate of competence initiation 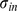, while 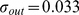. Speeds were determined over 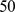 replicates and “shuffled” initial conditions were used. Error bars denote one standard error of the mean. Parameters are the same as in figure 2. Clonal initial conditions result in similar qualitative behavior and are explored in figure S8.

Based on observations from [14]–[16] (see Introduction), we model phenotypic switching by allowing cells to stochastically, and without memory, enter (exit) competence with rate 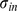 (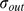) per unit time. In principle, the fraction (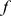) of cells expressing competence at any particular time is governed both by the switching rates (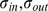) and the strength of selection for one phenotype over another. For our persistence model (see above) and experimentally motivated parameter values (see Methods), we find that selection only negligibly affects 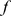 in our simulations (see figures S6 and S7). Thus, after a brief transient period, 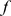 is well approximated by
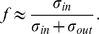
(3)This relationship roughly holds *experimentally* in *B. subtilis* [14]–[16] (see Methods). We measured the relationship between the fraction of competent cells (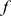) and the speed (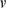) of adaptive evolution by varying 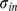 while holding all other parameters constant. Figure 4 (left) shows that when the rate of HGR (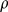) is large enough, 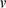 is fastest when a finite fraction (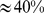 for these parameters) of cells express competence at a particular time. This supports our hypothesis that *phenotypic diversity for competence can be favored by natural selection, even in an unchanging environment*.

This striking result has a simple conceptual explanation. Increasing 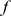 causes both an increased effective level of HGR, which accelerates adaptation (figure 2), and an increased generation time, which acts to slow down adaptation. The fact that these forces oppose one another presents the possibility that there exists a nontrivial 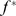 (i.e. 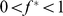) that strikes the optimal balance between cost and benefit. An additional piece to the puzzle, evident in figure 4, is that 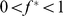 only when 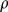 is large enough. This makes sense in light of the shape of figure 2, which becomes flatter with increasing 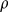. We can think of the parameter 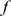 as tuning the effective rate of HGR (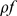) between the values zero and 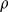. When 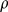 is small, only the steep portion of figure 2 is “accessible” as 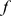 varies between zero and one, and the marginal benefit of increasing 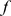 is large. Consequently, 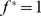 for small 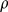 (figure 4). By constrast, for larger 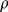, figure 2 becomes flat, and the marginal benefits of HGR become overwhelmed by the indirect cost of persistence for some 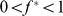.

The hypothesis that increased generation time is the relevant counterweight to the positive effects of HGR is supported by figure 4 (right), which considers the speed of adaptation *per generation* (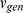). This measure naturally masks all generation time effects. We see that 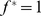 by this measure, i.e. the optimal strategy is for all cells to express competence. Of course, real competitions depend on fitness changes that occur in real time, and therefore 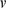, not 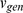 is the better measure of success.

### Direct Evolutionary Competitions Can Favor Phenotypic Switching between Competent and Vegetative Phenotypes

**Figure 5:**
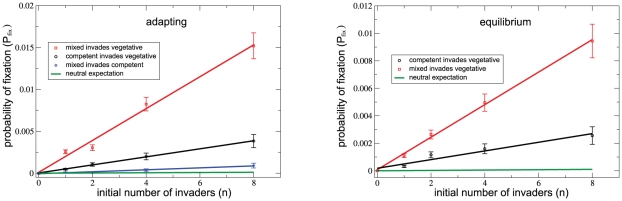
Mixed populations are competitively superior to purely competent ones, both during adaptation (left) and at mutation/selection/drift equilibrium (right). When invading vegetative residents during adaptation, each stochastically switching cell leaves an average of 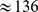 descendants: 4 times as many as competent invaders. Likewise, under equilibrium conditions, each stochastically switching cell leaves an average of 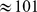 descendants: again, 4 times as many as competent invaders. Mixed populations are also able to directly invade competent residents during adaptation (blue diamonds, left) but not at equilibrium. The linear increase suggests that frequency-dependent selection is not operating (see main text). The neutral expectation represents 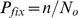, where 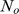 is the number of cells present at the beginning of competitions. 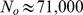 (left) and 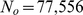 (right). Error bars denote one standard error. Each data point was derived from a number of replicate competitions greater than 10,000/(initial # invaders). Solid lines are the least squares linear fit. “Stochastically switching” populations had 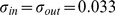, 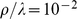). All other parameters are the same as above. Using 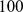-fold smaller mutation rates results in similar qualitative behavior for the adaptive case (left), and is explored in figure S10.

Above, we discussed the speed of adaptation, which measures evolutionary success at the *population level*. We now turn our attention toward direct competitions which measure evolutionary success at the *individual* level. We placed an initially rare “invader” in the context of a much larger “resident” population. For simplicity, we did not allow invaders to mutate into residents or vice versa. We did not observe stable coexistence between invaders and residents. Rather, after a sufficiently long period of time elapsed, the invader's lineage either went extinct or, occasionally, conquered the entire population (i.e. went to fixation). The probability of fixation (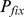) was then compared to the expectation under selective neutrality, which is simply the initial fraction of invaders (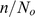). Figure 5(left) shows the results of competitions initiated as the resident population adapted up the fitness peak (see Methods). One set of invaders fully committed to competence whereas another (favored) set stochastically switched between the two phenotypes. We see that the fully competent invaders are favored, but the invaders that switch are even more highly favored. This supports our previous conclusion that stochastic switching is optimal during adaptation.

We also initiated competitions in which the population was *not* climbing the fitness peak but, rather, had already reached mutation/selection/drift equilibrium (see Methods). Figure 5 (right) shows that stochastically switching populations were more likely to conquer a vegetative resident than were purely competent cells. Unlike the adaptive case, mixed invaders never beat competent residents in equilibrium, which is unsurprising since competent populations have higher equilibrium fitness (figure 3). The competitive success of stochastically switching invaders can be rationalized by their optimal speed of adaptation (figure 4). However, we do not have a corresponding simple explanation for the advantage of stochastic switching in equilibrium. We hope to pursue this topic in future work.

To put the data from figure 5 in perspective, consider the expected number of descendants (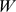) left by each invader, which equals 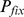 divided by the initial frequency of invaders. A neutral allele, of course, produces just one descendant (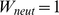). During adaptation and invasion of vegetative residents, stochastic switchers have 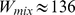, whereas for purely competent invaders, 
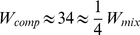
. Stochastic switchers have 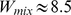 when directly invading competent residents. In the equilibrium case, 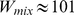 and 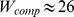, and so again 
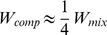
. Since each of these values is much larger than unity, they can be compared to the scaled selection coefficient usually denoted “Ns” in population genetics.

We anticipate that HGR will also confer a competitive advantage in other parameter regimes. Gordo and Campos [50] showed recently, via simulation, that in a mutation/selection/drift equilibrium context eukaryotic sex is most favored for mutations of intermediate strength– 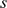 must be small enough to promote genetic diversity but not so small as to be invisible to selection. We expect this same logic to apply to our somewhat different model of bacterial transformation. Those authors, as well as others [48], also found an increasing advantage to sex as population size increased, but only in the regime where 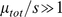. For 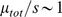 we expect the opposite trend to occur, since the effect of Muller's ratchet is strongest in this regime. In figure S10 we explore the case of 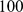 fold smaller mutation rates. In that case, mixed cells continue to invade adapting populations. However, in equilibrium populations with small mutation rates, we observed no fixations of invaders of any type, from which we conclude that the fixation probability of those invaders was less than or comparable to 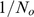.

To summarize, at the large mutation rates in figure 5, stochastically switching cells are competitively superior to purely competent cells in every scenario tested except when switchers invade competent cells in mutation/selection/drift equilibrium. These results suggest an interesting dynamic in which a purely competent population displaced from its fitness peak (by e.g. an environmental change) becomes susceptible to invasion by switching cells, but then later becomes susceptible to reversion to the purely competent strategy once adaptation ceases and equilibrium is regained.

As a final point, we consider whether our invaders exhibit “frequency-dependent selection,” as was recently reported in a simulation study [23] for the related case of invaders that perform HGR but not persistence. In order to determine whether frequency-dependent selection is operating, it is first necessary to articulate the behavior of a null model of “frequency-independent selection.” In the null case, each of 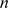 invaders would have an independent probability 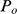 of fixating, and thus 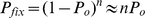 if 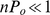. By contrast, frequency-dependent selection means that 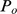 depends on 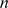. Figure 5 shows a clear linear increase of 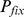 with 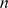, consistent with the null expectation. This linear trend continued for 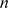 up to 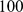 (for clarity, data not shown). The authors from [23] concluded that frequency-dependent selection was present, based (only in part) on their observation that out of 50 replicate trials, 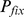 was large when competitions were initiated with a 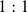 ratio of resident to invader, but small or zero when, say, a 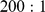 ratio was used. Our model would almost certainly exhibit this same qualitative behavior, but we emphasize that both our data and theirs is consistent with a simpler explanation, and neither data requires frequency-dependent selection. In order to fully explore frequency dependence in this system, one would need to measure 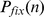 for all 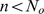 and examine whether this function can be fit by the frequency-independent model. In that regime, where invaders are initially abundant, HGR may well display some frequency dependence.

### Finite-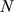 Deterministic Equations

Our proceeding results were based exclusively on computer simulations whose underlying Markov process cannot be solved analytically. Below, we develop a set of equations that approximate these simulations. The solutions display impressive qualitative agreement with the key conceptual findings discussed above, but the quantitative agreement is often weak (compare symbols and solid curves in figures 2,4). The utility of these equations is that they can be rapidly solved numerically, for arbitrary parameter values. This allows a qualitative exploration of regions in parameter space that are biologically relevant but consume prohibitively large amounts of CPU time. Below, we briefly present the finite-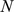 deterministic approach, the basis of which is treated in detail elsewhere [22], [36]. Table 1 summarizes the notation used. More detail can be found in Methods.

**Table 1:** Commonly used notation.

Symbol	Usage
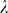	Number of genomic fragments (bits) in genome
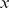	Fraction of 1's in a genome
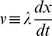	Speed of fitness increase
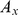	Number of vegetative (asexual) cells with fitness level 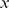
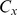	Number of competent cells with fitness level 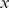
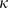	Population carrying capacity
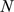	Total number of cells
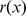	Intrinsic birth rate of vegetative cells
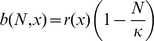	Actual birth rate of vegetative cells
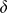	Death rate of vegetative cells
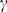	Factor by which birth and death are slower in the competent state
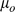	Deleterious ( 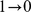 ) mutation probability per locus per replication
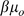	Beneficial ( 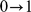 ) mutation probability per locus per replication
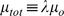	Total genomic mutation rate (assuming that all alleles are 1's)
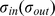	Switching rate into (out of) the competent phenotype
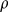	Recombination rate per genome (per time)

Parameters are denoted by Greek letters, whereas dynamical variables are denoted by Latin letters.

The basic goal of our finite-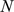 determistic equations is to dynamically describe the number of cells carrying a given fraction (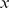) of “ones” in its genome. This is determined by the processes of birth, death, mutation, and HGR, which we will consider in turn. The birthrate (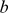) depends on both 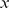 and the total number of cells (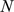), via a simple logistic factor (see Methods). By contrast, the death rate (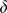) is a constant in our model. Thus, if we neglect stochastic fluctuations and temporarily omit mutation and HGR, then the number (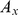) of vegetative (asexual) cells with a given fraction of “ones” (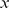) is given by
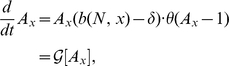
(4)where 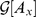 is the “growth operator” acting on the population 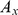. The term 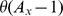 is the Heaviside step function which simply equals one if 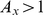 and zero otherwise. The purpose of this “cutoff factor” is to *heuristically* incorporate finite number fluctuations and prevent fractional numbers of very fit individuals from growing extremely quickly and dominating the dynamics [58], [59].

Now let us consider mutation in isolation from birth, death, and HGR. Upon birth, each of 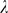 loci can be “flipped” from 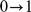 or vise versa. Deleterious mutations occur at a rate 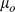 at each of the 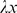 loci carrying a “one.” Similarly, beneficial mutations occur with rate 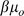 at each of the 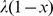 loci carrying a “zero,” where 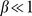 reflects the preponderance of deleterious over beneficial mutations. These mutations result in a flux of cells between fitness level 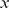 and its neighboring fitness levels (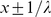). Thus, mutation acting in isolation can be represented by as 
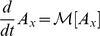
, where the mutation operator 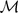 is given by
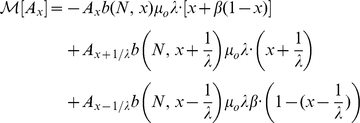
(5)This equation merely expresses single beneficial and deleterious mutations occurring between neighboring “fitness classes.” We neglect the chance of more than one mutation occurring during a single replication event in these equations (but not in the simulations).

The growth and mutation operators can be combined in a single equation that has proven qualitatively successful in describing asexual evolution dynamics [59]: 
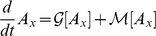
. This is essentially a quasi-species equation [60], [61], except for two non-traditional features: (i) distinct sequences are binned according to their fitness value (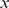) and, (ii) the presence of the cutoff factor 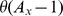.

We now include recombination, which is much more difficult to model. When an HGR event occurs, a consequential genomic change happens only if the donor allele differs from that of the acceptor. The frequency of such events is deeply related to the probability that two randomly chosen cells differ at a particular locus under selection, often referred to as the “heterozygosity.” Since we are unaware of any rigorous method to calculate this quantity in our finite 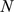, multi-locus setting, we invoke a strong but useful approximation. Specifically, we assume that *every genotype containing* 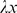 *ones is uniformly represented in the population at all times*. In other words, we assume a maximal level of genetic diversity within each fitness class [62]. Under this assumption, the probability that a “one” is chosen as the donor allele at a particular locus is simply the probability that a “one” is the donor at *any* locus, i.e. the mean fraction of “ones” in the population (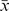). We assume that this is true regardless of 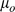.

Thus, we can construct a recombination operator 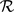:
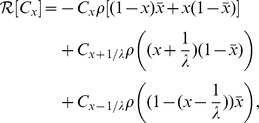
(6)where 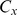 denotes the number of competent cells with fraction 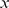 of “ones.” In figure S1 and text S1 we construct a modified recombination operator that represents an external DNA pool loaded with excess deleterious mutations. This modification does not change our essential results.

After assembling these dynamical ingredients, including the persistence factor 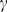, and allowing for phenotypic switching between 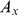 and 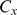, we obtain the coupled set of equations
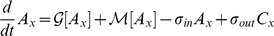
(7)

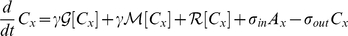
(8)


Solutions to equations 7,8 are plotted as solid curves in figure 2,4. We see that the finite-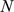 deterministic equations reproduce the qualitative behavior of simulations, but, on a quantitative level, they always overestimate the speed of adaptive evolution (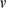). Quantitative agreement is strongest when “shuffled” initial conditions were used, because the recombination operator (equation 6) assumes this extreme amount of neutral diversity within each fitness class. However, this degree of diversity cannot be maintained by finite populations, resulting in an overestimate of the effects of HGR and hence the speed 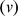 as well. The quantitative disagreement is large when simulations were founded with a single clone because, over the timescale of the population climbing the fitness peak, the population is unable to generate the neutral diversity assumed by the recombination operator. However, our most important qualitative conclusion– that stochastic switching in and out of competence is evolutionarily optimal– remains true even if clonal conditions are used (see figure S8). In the artificial case that that the carrying capacity 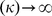, every possible genetic sequence exists for all 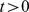, and recombination confers only a very small advantage. Consequently, the optimal strategy in this case is for no cells to express competence (pure vegetative growth) (see figure S9).

Two very recent studies [37], [63] develop a stochastic analytic approach to the dynamics of adapting sexual populations. Those authors calculate the fixation probability of new beneficial mutations as they continually recombine into different genetic backgrounds, then relate this fixation probability to the speed of adaptation. The stochastic nature of these calculations is an improvement on our essentially deterministic equations, although, unlike our study, they neglect deleterious mutations. In addition to eukaryotic recombination, Neher et al. [37] also address a bacterial transformation model similar to ours. They consider an infinitely long genome where the number of segregating mutations is set by the balance between random drift, the rate of recombination, and the strength and production rate of beneficial mutations. Recombination is assumed to shuffle these segregating mutations into all possible combinations, consistent with a Gaussian fitness distribution. These assumptions are superficially reminiscent of those we employ in constructing our recombination operator (equation 6). However, the assumptions are in fact quite different. Whereas they assume the occupation of all fitness classes consistent with combinations of newly segregating beneficial mutations, we assume the reverse– namely the occupation of all genotypes consistent with a given fitness distribution.

Whereas our methods overestimate the speed of adaptation from simulations, theirs yields an underestimate to the eventual steady state velocity. Generally, their assumptions are much more reasonable than ours for the problem of steady-state adaptation, though ours may better approximate scenarios following a sudden environmental change, in which previously neutral or mildly deleterious polymorphisms are initially present [62] (e.g. our “natural initial conditions”). Furthermore, it is worth reiterating that the eventual steady state considered in those studies is reached only after an extremely long transient, as evidenced by the sensitivity to initial conditions in figure 2.

## Discussion

We constructed a model of bacterial competence that includes both recombination throughout the entire genome and also a persister feature to the competent state. We found that HGR is indirectly favored in this model, even without the presence of epistasis (see below). Persistence, on the other hand, is indirectly disfavored during adaptive evolution in our model. When we coupled persistence and HGR by allowing cells to stochastically switch between competent and vegetative phenotypes, we found that the optimal differentiation strategy often entailed a mixed population of the two phenotypes, reflecting a tradeoff between HGR and persistence. Optimality of the mixed strategy during periods of adaptation (i.e. positive selection) was rather robust to parameter values (figures S1, S2, S3, S4, S8, S9, S10). Besides the case of positive selection, cells employing the mixed strategy also successfully invaded resident populations in mutation/selection/drift equilibrium under most scenarios. However, there were some ambiguities and exceptions (see Results). The more robust advantage to the mixed strategy during positive selection may reflect the fact that, in many species, the competence system is activated during stress [12], i.e. when the population is displaced from its fitness peak. Below, we discuss this article's limitations and relate it to previous studies of recombination and phenotypic diversity.

### Boom-Bust Dynamics and Bet Hedging

Our birth and death dynamics assume that the overall population size is (at least approximately) constant. By contrast, previous models of persistence [6], [21], [64] focus on cycles of boom and bust resulting from environmental changes. During booms, the population expands and non-persisters exponentially outgrow persisters. Conversely, during busts, non-persisters die exponentially while the persisters remain intact.

A modified version of these dynamics was recently applied to competitions between strains of *B. subtilis* that possess normal competence genes (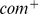) and a strain with a disabled competence system (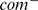) [6]. Since a subpopulation of 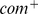 cells expresses competence, which includes persister effects, this strain is favored over the 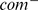 strain during busts (mediated by antibiotics) but disfavored during booms (access to fresh media). This observation was supported by experiments those authors performed on *B. subtilis*. In stark contrast to our model, theirs does not include *homologous* recombination (HGR). Rather, they include the effect of recombination only by allowing the occasional acquisition of strongly beneficial genes (e.g. antibiotic resistance) from a truly exogenous source, e.g. another species. Furthermore, they do not address the potential optimality of mixed competence expression.

Nevertheless, boom and bust dynamics could, in principle, be relevant to understanding mixed competence expression via a “bet-hedging” mechanism [19], [21], [64], [65]. One straightforward way for a population to cope with uncertainty is by sensing the environment and then responding with the appropriate phenotype. A different strategy, known as “bet-hedging,” is to *blindly* and simultaneously express diverse phenotypes. The obvious cost to this strategy is that some cells invariably express the inappropriate phenotype. Interestingly, this cost is minimized by a level of diversity, and underlying switching rates, that mirror the frequency of environmental change [21], [64]. Bet-hedging can be favored over a sense-and-response strategy when environments change infrequently and the sensing apparatus imposes a large enough cost [21].

In the context of *B. subtilis*, bet-hedging implies that the cell is blind regarding whether the environment is suitable for competence expression. However, this seems inconsistent with the well known fact that competence in *B. subtilis* is a tightly regulated stress response [12] to particular environmental cues. Thus, bet-hedging seems unlikely to explain diverse competence expression because *B. subtilis* pays *both* the diversity cost and the sensing cost. Furthermore, the competence system involves a large number (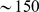) of genes [66], suggesting that it did not evolve primarily as a persistence system, which would presumably require far fewer genetic components. The apparent failure of bet-hedging explanations in this context motivates the central hypothesis of this article– that diversity in competence expression is itself optimal in a population-genetic sense.

A recent study [16] used the bet-hedging framework to address a related but distinct aspect of competence expression in *B. subtilis*. In particular, they investigated the optimal distribution of competence duration times, finding that a broad distribution is best able to hedge against uncertain concentrations of extracellular DNA. Their underlying assumption is that the ideal strategy for the cell is to remain competent for long enough to encounter sufficient DNA, and then return to vegetative growth. The purpose of our article is precisely to understand the basis of this assumption.

### Previous Population Genetic Studies

Our results have some bearing on the evolutionary advantage of sex and recombination. There is an enormous amount of literature covering this topic, most of which is oriented toward diploid eukaryotes. Although there are non-trivial differences between meiotic crossing over and bacterial transformation, models of the former provide insight to competence. Below, we touch upon some of this literature.

The essential effect of recombination is to reduce the correlations between alleles at different loci. Without these correlations, recombination can have at most a tiny effect. Correlations among loci are known as “linkage disequilibrium” (LD), which can have several origins. One source of LD, known as the “Fisher-Muller effect,” [45], [51] occurs in adapting populations in which more than one beneficial spreads (i.e. “segregates”) simultaneously. Our parameter values correspond to this regime. In asexual populations, these concurrently spreading beneficial mutations most likely originate (and remain) in different backgrounds. Therefore, in the absence of recombination, the presence of one beneficial mutation is anti-correlated with the presence of the other, i.e. LD is negative. Recombination brings the mutations together in a common chromosome, which pushes LD closer to zero and accelerates adaptive evolution. The Fisher-Muller effect underlies the advantage to recombination seen in figures 2 and 5(left), and also in previous studies of HGR [22], [23].

When beneficial mutations are not common enough to generate the Fisher-Muller effect, recombination can be favored in infinite populations if “synergistic epistasis” is present [67]–[70]. Synergistic (also called “negative”) epistasis means that each additional deleterious mutation has a larger effect than those which preceded it. Consequently, sequences carrying multiple deleterious mutations are under-represented in the population, as compared to the case with no epistasis. Thus, synergistic epistasis generates negative LD between deleterious mutations. Recombination decreases the extent of this LD and, under certain restrictions on the strength of epistasis [69], [70], can be favored. Importantly, each of these studies predict that if epistasis is absent or “antagonistic,” LD will not be negative and recombination will not be favored by evolution. While correct in the infinite 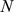 limit, this prediction does not apply to moderately size populations, as can be seen in figures 3 and 5(right) and previous studies [48], [50]. In fact [48], [50], show an *increasing* advantage to recombination as 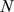 increases from 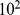 to 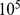 (when 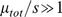). We expect the 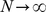 prediction to hold when the number of cells is much larger than the number of combinations of loci under consideration (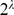). When the number of genotypes is large (e.g. 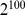 in our simulations), the 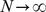 theory [67]–[70] may not be a good approximation for any realistic value of 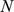.

Three previous studies [23]–[25] explicitly consider HGR in bacteria, but not phenotypic switching into competence. Redfield and co-workers studied the equilibrium level of fitness achieved by infinite populations [24], [25], finding that synergistic epistasis is required in order to confer an equilibrium advantage to HGR, in accord with (and subject to the same limitations as) the aforementioned 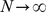 theory. However, they do not consider the important case in which beneficial mutations are available and the population is adapting (i.e. positive selection). A major strength of that study is that they consider interesting issues that our work largely neglects, such as recombination of genes responsible for HGR and the possibility that alleles in the extracellular pool may tend to be loaded with deleterious mutations (although see figure S1 and text S1). Future work could reconsider these important complications in our stochastic, finite 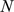 context. Recently, Levin and Cornejo [23] devised an HGR model that bears many similarities with ours, although they do not consider phenotypic switching. In rough agreement with our results, those authors found that HGR accelerates adaptation and that HGR can invade asexual residents (although, see commentary surrounding frequency-dependent selection in Results and also figure 5). The most important difference between our approach and theirs is that they included only five loci with small fitness effects. Each of these loci had a very small mutation rate (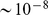), suggesting that they represent perhaps 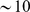 nucleotides each. Thus, their approach neglects the vast majority of genotypic diversity present throughout the rest of the genome. This is especially important in the context of recombination because the frequent mutations in this region generate sequence diversity upon which recombination will act.

### Mechanisms of Cell Death

A prominent feature of our model is cell death during competence inducing conditions. Cell death is usually not explicitly measured during laboratory experiments unless killing agents (e.g. antibiotics) are applied. However, in natural populations, it seems quite reasonable to assume that birth and death balance, in what has been referred to as “long-term stationary phase” [71]. Additionally, in *B. subtilis*, there are complications that interrelate competence, sporulation, and cell death. Competence and sporulation are distinct stress responses in *B. subtilis*, but they are often activated during the same conditions [12], [72]. Spore formation involves asymmetric cell division in which the eventual products are a spore and a lysed non-competent cell (see, e.g. [73] for a review of sporulation in *B. subtilis*). Also, recent experiments [74], [75] demonstrate “cannibalism” in which sporulating cells secrete factors that kill non-sporulating cells. The related phenomenon of “fratricide” occurs during competence induction in *S. pneumonia*. Together, these observations suggest that cell death, particularly among non-competent cells, is both important and commonplace under conditions relevant to the evolution of competence. Careful treatment of these phenomena, and their interrelationships, is beyond the scope of the relatively simple model presented here, but could be pursued in future work.

### Limitations of Current Work

In this article we make several assumptions that could be relaxed in future work. First, our model neglects sporulation, which may be inherently coupled to the competence system in *B. subtilis* [72].

Secondly, we have not directly represented the genes responsible for recombination in 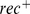 cells (i.e. a “modifier locus”). Since bacterial transformation is non-reciprocal, this modifier locus can exchange itself for a non-functional homologue in the extracellular pool, thereby becoming 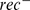. However, the reverse process required to replenish the number 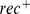 cells cannot occur, and thus the number of 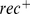 cells should decrease under this influence. This process cannot alter our results concerning the rate of adaptation (figure 2,4) because all cells in those populations carry the modifier locus. However, this effect will to some extent impact our results concerning competition experiments (figure 5). This issue should lead to an effective selection coefficient 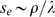 against 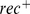 cells. Based on our parameter estimation (see Methods), this implies a 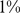
–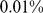 effective disadvantage to 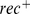. Since we have estimated 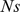 for recombining invaders (figure 5), we can very roughly estimate a selection coefficient of 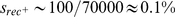 in favor of 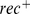. This indirect benefit may or may not be sufficient to overcome the 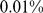
–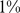 decay caused by non-reciprocal exchange. Of course, it is important to remember that genes enabling a 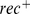 phenotype are obviously somehow maintained in many real bacterial populations. It has been pointed out by other researchers that many genes enabling 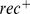 have other important functions, and that the capacity for HGR might only be maintained as a by-product (see [76] for a review). In this article, we do not take a position on whether HGR is the dominant reason that these genes exist. Exploration of that topic requires detailed experimental knowledge of the pleiotropic effects of these genes, as well as estimates of their fitness consequences. Rather, we have merely isolated, quantified, and attempted to deepen understanding of the population genetic aspects to competence. Relative to some previous studies [24], [25], our stochastic treatment reveals that HGR can be favored strongly and broadly (e.g. without epistatic effects).

Thirdly, we have assumed that competence does not entail a direct fitness (dis)advantage. Although we suspect that our broad qualitative conclusions will remain true if competent invaders are assigned a sufficiently small direct birth/death penalty, future work could quantify the maximum size of this handicap. Along these same lines, one could allow the overall population size to either grow (directly favoring the vegetative phenotype) or shrink (directly favoring the persistence phenotype).

Fourthly, we have assumed that all mutations have the same effect on fitness, which is obviously not true in real populations. A step toward greater realism could be made by incorporating a set of loci that become lethal if mutated.

Fifthly, our fitness function (equation 1) is non-epistatic; in other words, different loci make independent contributions toward organismal fitness. This is a significant assumption, given the prominent role of epistasis in theories of the evolution of sex/recombination. However, our non-epistatic assumption is conceptually neutral in that it does not “automatically build in” the result that HGR is indirectly favored by evolution. Indeed, as discussed above, 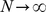 population genetic theory predicts neither an advantage nor a disadvantage to recombination in the absence of epistasis. Additionally, the experimental data is mixed and inconclusive (e.g. [77]–[79]) regarding whether synergistic, antagonistic, or no epistasis predominates between loci in real bacterial genomes. Given this set of facts, our assumption of no inter-locus epistasis seems fair. Nevertheless, future work could investigate our model under various epistatic fitness functions.

Finally, we point out an empirical shortcoming of our theory: Figure 4 predicts that the speed of evolution is optimized when 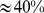 of cells express competence, which is significantly larger than the 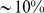 observed in the laboratory. This *quantitative* discrepancy could be due to any or all of the limitations listed above.

### Experimental Predictions

A fundamental prediction of our theory is that, if total population size is approximately constant, cells which stochastically switch between the competent and vegetative phenotypes will prevail in competition experiments against otherwise isogenic cells that are either 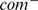 or that fully commit to competence. Süel et al. demonstrated experimentally that the switching rates (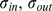) can be independently tuned by manipulating the basal expression rates of 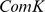 and 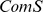, respectively [15]. Thus, in principle, the fraction of competent cells can be experimentally adjusted (equation 3) while holding constant the time spent in competence. Although conceptually straightforward, there are potential complications to these experiments. First, both serial-passage and chemostat protocols discard excess cells indiscriminately, resulting in the same “death” rate for both the competent and vegetatively growing phenotype. An alternative that circumvents this problem is long-term batch culture [71]. However, in this case metabolic waste products accumulate and the environment is not constant. A second complication to this suggested experiment involves the possibly bizarre behavior of cells engineered to fully commit to competence. Recall that normal *B. subtilis* cells elongate while expressing competence, then fragment into 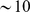 daughter cells only upon exit from competence ([14], supplementary movies). Thus, if exit never occurs, these cells might not ever divide. In this case, our simple, continuous growth model completely misrepresents the strange behavior of the engineered cells. However, this does not at all change our central conclusion that phenotypic diversity for competence is evolutionarily favored over total commitment to competence, since non-dividing cells will obviously lose the competition.

Although we constructed our model with the behavior of *B. subtilis* in mind, our assumptions and conclusions may also be appropriate for a broader class of naturally transformable bacteria. First, we followed Johnsen et al. [6] in assuming that competent cells replicate slowly compared to vegetatively growing cells. As those authors point out, this effect has been observed in *B. subtilis* [4]–[6] and *S. pneumoniae* [80] but has not been observed, nor specifically investigated, in other species. Along with those authors, we predict that in other naturally transformable species, phenotypically competent cells will also replicate slowly compared to vegetatively growing cells. Additionally, we predict that, in a range of naturally competent species, phenotypically competent cells will die more slowly than vegetatively growing cells under natural competence stimulating conditions. More strikingly, we also predict that, in other species as well as *B. subtilis*, observations of single cells over relatively long time-scales will reveal phenotypic switching to a faster growing, non-recombining phenotype.

### Concluding Remarks

Evolutionary modeling on a genome-wide scale necessarily entails many rough and abstract assumptions. However, the staggering complexity of real biological systems does not necessarily preclude insightful contributions from abstract population-genetic models. In this article we have included many ubiquitous features such as multiple loci, genetic linkage, and both beneficial and deleterious mutations. All of our parameter values are based on experimental estimates. The conceptual basis of our conclusions is rather simple: Homologous recombination (HGR) by itself is evolutionarily favored, but this advantage is offset by a reduced replication rate. Slower replication may be unavoidable if a cell is to avoid DNA errors introduced by the HGR process. The optimal balance between cost and benefit is achieved by allowing the novel recombinant genotypes created by HGR during competence to cycle back and later be expressed in rapidly growing vegetative cells. Because competence initiation and exit are stochastic and asynchronous, these cycles result in heterogeneous competence expression throughout the population. The link between persistence and competence plays a crucial role in our model. Besides *B. subtilis*, there is also evidence for this link in *S. pneumoniae* [80]. To our knowledge, this connection has not been observed, nor specifically investigated, for other bacterial species. In all likelihood, there are many counterexamples to the competence-persistence link– these signal violations of our simple model assumptions and could point toward interesting biological questions.

## Supporting Information

Figure S1According to our finite-N deterministic approach, HGR can accelerate adaptation, even when it draws from a DNA pool loaded with deleterious mutations. The negative impact of the contaminated pool becomes more pronounced with increasing levels of HGR (left). Mixed competence expression continues to be optimal, even with the contaminated pool (right). Parameters are 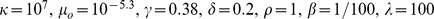.(0.50 MB EPS)

Figure S2The effect of HGR depends on the strength of selection (via 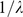). 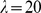 corresponds to a 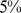 selection coefficient whereas 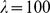 corresponds to 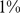. When standing genotypic diversity is extremely large (shuffled initial conditions, left) strong HGR causes both strong and weak beneficial alleles to accumulate at the same rate. However, we note that the speed of *growth rate* increase is equal to 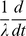 “ones”. Thus, in this sense, populations with 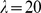 always evolve faster because each mutation has a greater effect on growth rate. Our finite-N deterministic equations (solid lines) provide a decent approximation of this regime. When clonal initial conditions are used (right), there is less diversity on which HGR can act, and the population with stronger mutations (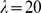) always evolves faster. Also notice the decrease in 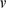 for large 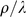 on the right panel: non-reciprocal bacterial transformation can remove precious rare beneficial cells in this regime. Recent theoretical work by Neher et al. [37] predicts that HGR becomes strong enough to significantly increase 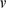 when a beneficial allele recombines many times as it ascends to high frequency in the population. This means that 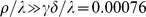, i.e. 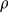 (and not 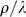) reaches a critical value. By contrast, our data suggests that the effect of HGR is governed most clearly by 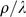 (and not 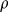) for both data sets. This discrepancy could be because those authors consider the eventual steady state rate of adaptation, which may not be reached in these simulations. Clearly this issue requires more attention in future work. Parameters are 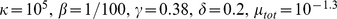 (all data had the same mutation rates *per genome*.) Speeds were measure between 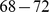 “ones” when 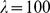 and between 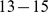 “ones” when 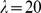.(0.48 MB EPS)

Figure S3Speed of adaptation increases with population size (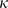). Our finite-N deterministic equations perform better at larger 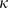 and when standing genotypic diversity is large (shuffled initial conditions, left). Population size has a larger effect on speed of adaptation when there is less genotypic diversity (clonal initial conditions, right). Notice the different scales on the left and right panels. Parameters are 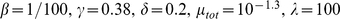. Speeds were measured between 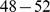 ones (small populations could not always achieve higher fitness values due to Muller's ratchet).(0.48 MB EPS)

Figure S4The effect of mutation rate depends dramatically on initial conditions. When standing genotypic variance is large, recombination dominates the adaptation process (left). Our finite-N deterministic equations (solid lines, left) predict that HGR will dominate the dynamics, and that mutation will have an almost undetectably small effect. By contrast simulations (left) show a somewhat larger, retarding effect to mutation. In the opposite extreme, when standing genotypic variance is small (clonal initial conditions, right), mutation has a large, accelerating effect on the speed of adaptation (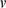) and the efficacy of HGR. HGR enabled a 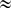
2-fold increase in 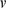 for small mutation rates, and a 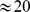-fold increase for large mutation rates. Notice that strong HRG can decrease 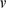 when genotypic diversity is small (right). Parameters are 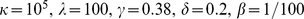. Speeds were measured between 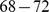 ones.(0.49 MB EPS)

Figure S5HGR does not confer an equilibrium advantage to fitness when 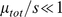. However, figure 3 from the main text shows that a substantial equilibrium advantage can occur for 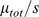 as small as 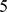. We suspect that HGR may confer an equilibrium advantage even for the realistically small mutation rates considered here, if more weakly deleterious mutations and/or smaller population sizes are used. Parameters are 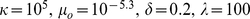 (i.e. 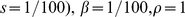.(0.57 MB EPS)

Figure S6The fraction of competent cells (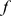) is governed by the switching rates 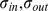
according to the formula 
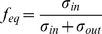
, after a brief transient. Single randomly chosen trajectories using different values of 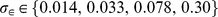 are plotted. Parameters are 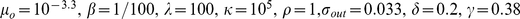.(0.02 MB EPS)

Figure S7The total number of cells (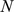) increases with fitness in a predictable way. Ignoring mutation, recombination, and phenotypic switching, the deterministic equation for the total number of cells is 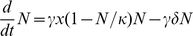. Setting 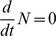 leads to 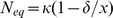, which is an adiabatic approximation for 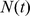. This result is plotted along with single randomly chosen trajectories using different values of 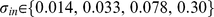. Parameters are 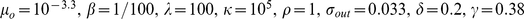.(0.05 MB EPS)

Figure S8This is the same as figure 4 from the main text, but the simulation points were derived from clonal initial conditions. These initial conditions lead to much smaller speeds of evolution, but the key qualitative behavior remains the same. In particular, mixed competence expression maximizes 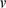 when 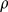 is large enough. Parameters are 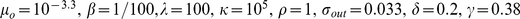.(0.09 MB EPS)

Figure S9More solutions to the finite-N deterministic equations. All solutions are essentially independent of the mutation rate for the parameters investigated here (small mutation rate case plotted only). Here we see that the level of competence heterogeneity (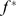) that maximizes 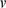 is a decreasing function of 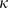. In the deterministic limit (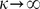), the optimal strategy is for all cells to grow vegetatively. Parameters are 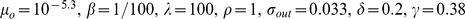.(0.09 MB EPS)

Figure S10A mixed competence strategy is competitively optimal in adapting populations with realistically small mutation rates. When invading vegetative populations, mixed invaders each left 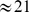 descendants (red). When invading competent residents, each invader left 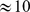 descendants. Thus, the advantage to mixed invaders is less impressive but still substantial for the small mutation rates considered here. Unlike the high mutation rate case, purely competent cells were not able to invade here. This is likely because the benefits of HGR do not compensate for the cost of persistence when only a small number of beneficial mutations are segregating. When mixed or competent cells invaded vegetative residents at mutation/selection/drift equilibrium, no fixations were observed (data not shown). Parameters are 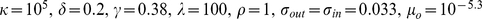.(0.07 MB EPS)

Text S1 Finite-N deterministic equations for external DNA pool loaded with deleterious mutations.
(0.02 MB PDF)
