# Supplementary material for: Optimal Strategy for Competence Differentiation in Bacteria
Source: PLoS Genet. 2010 Sep 9;6(9):e1001108. doi: 10.1371/journal.pgen.1001108 (PMC2936531; doi:10.1371/journal.pgen.1001108)
Supplement: Text S1 — Finite-N deterministic equations for external DNA pool loaded with deleterious mutations. (0.02 MB PDF) [file pgen.1001108.s011.pdf]

Optimal strategy for bacterial competence: supplementary text

We modify our recombination operator to take into account excess deleterious mutations present in the extracellular DNA pool. In this case, the probability that the "donor" allele is beneficial is no longer $\bar{x}$ but rather $\bar{x}(1-\epsilon/\lambda)$, where ϵ is the average excess number of deleterious mutations. Thus, the recombination operator becomes

$$\begin{aligned}\mathcal{R}[C_x] = & - & C_x\rho[(1-x)\bar{x}(1-\epsilon/\lambda) + x(1-\bar{x}(1-\epsilon/\lambda))] \\ & + & C_{x+1/\lambda}\rho\left((x+\frac{1}{\lambda})(1-\bar{x}(1-\epsilon/\lambda))\right) \\ & + & C_{x-1/\lambda}\rho\left((1-(x-\frac{1}{\lambda}))\bar{x}(1-\epsilon/\lambda\right). \qquad (S1)\end{aligned}$$

Like mutation, recombination now has a deleterious bias. Despite the bias, HGR, like mutation, can still accelerate adaptation as long as it does not operate too strongly. We can approximate the effect of the contaminated pool as increasing the deleterious mutation rate while decreasing the beneficial mutation rate, each by amounts $\sim \rho\epsilon/\lambda$. Since HRG is typically strong compared to mutation, these terms represent large perturbations to the mutation rate. In Fig.S1, we plot solutions from our finite-N deterministic equations (Eqs. 5,6 from main text). We see that our key results– that HGR can accelerate adaptation and that mixed competence expression is optimal– still hold even with contaminated extracellular DNA. Heuristically this is because, as long as the deleterious mutation rate is not too large, v is determined mostly by the fittest individuals near the leading edge ("nose") of the distribution. However, we anticipate that the additional effective deleterious mutations will be significant in a competitive context where more than one strain is present. We hope to address this issue in future work. To fully treat the issue of contaminated extracellular DNA, simulations should be done that circumvent the limitations of our finite-N deterministic equations. These proposed stochastic simulations require a more detailed model for how fitness (inversely) relates to the contribution made to the extracellular pool.
